# The inclusion of mobilisation with movement to a standard exercise programme for patients with rotator cuff related pain: a randomised, placebo-controlled protocol trial

**DOI:** 10.1186/s12891-020-03765-6

**Published:** 2020-11-12

**Authors:** Rafael Baeske, Toby Hall, Marcelo Faria Silva

**Affiliations:** 1 Science of Rehabilitation programme at Universidade Federal de Ciências da Saúde de Porto Alegre, Rua Sarmento Leite, 245, Porto Alegre, Rio Grande do Sul CEP 90050-170 Brazil; 2 São Leopoldo, Brazil; 3 School of Physiotherapy & Exercise Science, Curtin University, Kent Street, Bentley, Western Australia 6102; 4 Department of Physical Therapy, Universidade Federal de Ciências da Saúde de Porto Alegre, Rua Sarmento Leite, 245, Porto Alegre, Rio Grande do Sul CEP 90050-170 Brazil

**Keywords:** Musculoskeletal manipulations, Mobilisation with movement, Shoulder pain, Rotator cuff, Exercise

## Abstract

**Background:**

Rotator cuff related pain (RCRP) is one of the most common sources of musculoskeletal shoulder pain affecting the general population. Conservative treatment, in the form of exercise, is considered the first line approach, nonetheless, improvements seem to be modest. One therapeutic modality that might be an adjunct to the treatment of this condition is mobilisation with movement (MWM). MWM is a pain-free manual procedure that targets restricted and painful movements, commonly seen in patients with RCRP. The purpose of clinical trial is to determine whether MWM with exercise has benefits over sham MWM with exercise in RCRP.

**Methods:**

A randomised, sham-controlled trial of 70 adults complaining of RCRP will compare the effects of MWM combined with exercise over sham MWM with exercise. Participants will be allocated to one of two groups: exercise and MWM (EG) or exercise and sham MWM (CG). Two weekly individual treatment sessions will be conducted over five weeks. All assessments will be performed by a blinded assessor. Primary outcome measures will be the shoulder pain and disability index (SPADI) and the numeric pain rating scale (NPRS), assessed at baseline, discharge and one-month follow-up. Secondary outcome measures will be active range of motion, self-efficacy and the global rating of change scale. The analyses will be conducted considering a statistically significant *p*-value ≤0.05. Normality will be assessed with the Kolmogorov-Smirnov test and homogeneity with the Levene’s test. For the primary outcome measures (SPADI and NPRS) and self-efficacy, a 2 × 3 ANOVA with treatment group (EG versus CG) and time (baseline, end of the treatment and follow-up) factors will be performed. Separate 2 × 2 ANOVA will be used for range of motion (baseline and end of the treatment). Global rating scale of change analysis will be conducted using descriptive statistics. Intention-to-treat analysis will be adopted.

**Discussion:**

As there is a paucity of longitudinal studies investigating the use of MWM in patients with RCRP, this study will help to better understand its role together with a structured exercise programme.

**Trial registration:**

Clinical Trials Registry number NCT04175184. November, 2019.

## Background

Shoulder pain is one of the most common sources of musculoskeletal pain that affects up to 20% of the population [1]. Importantly, approximately 40% of people complaining of shoulder pain will still be symptomatic six months after onset [2]. Rotator cuff related pain (RCRP) or non-specific shoulder pain is a term that includes a diversity of shoulder conditions known as: subacromial impingement syndrome, rotator cuff tendinitis/tendinopathy, rotator cuff tear, and bursitis [3, 4]. The use of a broader term is useful as the diagnostic accuracy of special orthopaedic tests have been widely criticised and are unable to identify pathognomonic sources of symptoms in people presenting with shoulder pain [5–7]. Additionally, even though diagnostic imaging is capable of identifying pathology in patients with rotator cuff related pain, correlation of these findings with the clinical presentation is questionable [8–11].

Physiotherapy has an important role in the management of rotator cuff related pain, and exercise is the main therapeutic approach when considering pain and functional restriction [12–14]. However, the improvements seem to be modest [12, 15]. A recent update of systematic reviews has suggested that adding manual therapy to exercises might offer superior short-term decrease in pain [16]. However, this finding was based on few studies with low quality level.

Mobilisation with movement (MWM) is a musculoskeletal treatment approach that focuses on improving active pain-free range of motion [17]. One of the main cardinal signs in patients suffering from rotator cuff related pain is pain on active movement. MWM incorporates a passive glide force produced by the clinician, followed by an active movement executed by the patient. Different studies have suggested positive effects of MWM over a sham procedure in patients complaining of shoulder pain [18–20], while other studies reported no such effects [21, 22]. Several methodological aspects might have influenced this discrepancy in results, such as population studied, dosage and type of MWM utilized, as well as follow-up period and outcome measures. Of particular interest here is the fact that all studies that have investigated the use of MWM in patients with shoulder pain, utilized only one form of MWM. This aspect does not explore all MWM possibilities for patients with shoulder pain [17]. Consequently, the use of MWM in patients with rotator cuff related pain deserves greater investigation. Therefore, the purpose of this study is to explore the effects of MWM applied pragmatically, reflecting usual clinical practice for this form of musculoskeletal disorder management.

## Methods

### Objectives

Due to the uncertainty in MWM effectiveness for shoulder pain, the current research aims to explore the inclusion of MWM to a 5-week exercise programme in patients with rotator cuff related pain on different functional outcome measures and pain. Additionally, a comparison will be made with a previously used sham MWM [18] to account for contextual effects of treatment procedures [23]. Furthermore, we will conduct different secondary analysis (to be published separately) exploring the effects of the interventions applied (MWM and sham MWM) on pain pressure threshold in order to verify whether the interventions used have different mechanisms of action. A further aim of this study to be published separately, is to evaluate expectation on treatment outcome, which will be investigated at baseline and during the third week of treatment.

### Trial design

This randomized, placebo-controlled, parallel study design will be conducted in two different sites with data collection at baseline, after the treatment period and at one month follow-up. The study was designed following the standard protocol items for randomized interventional trials (SPIRIT) and the results will be reported in accordance with the consolidated standards of reporting trials (CONSORT) guidelines for randomized trials [24].

### Study settings

**Fig. 1:**
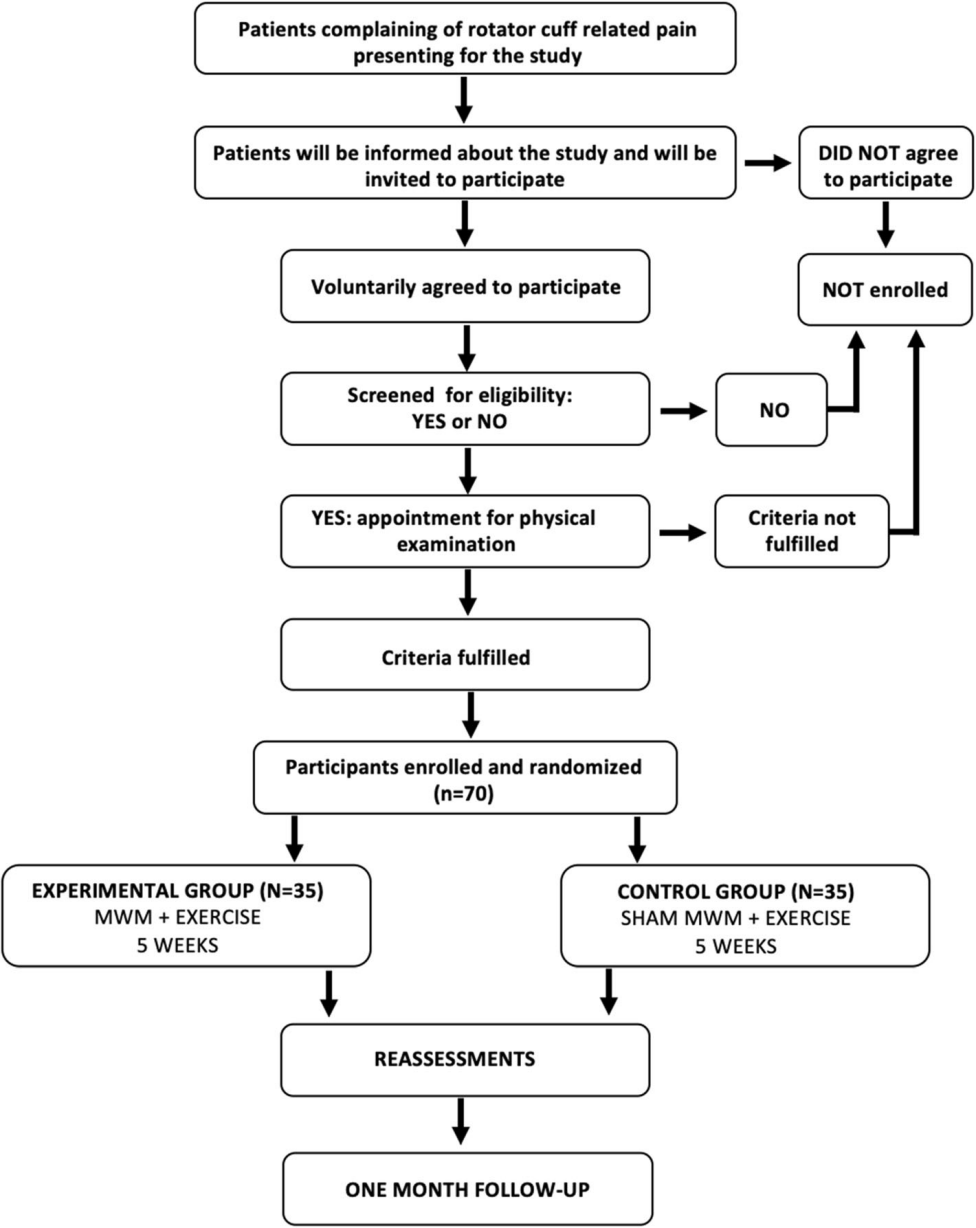
Recruitment process and flow through study

The study will occur in two different locations, at the physiotherapy laboratory 1 at *Faculdades Integradas de Taquara* and a private practice (*Clínica Albrecht*). The Recruiment process and flow through study is depicted in Fig. 1.

### Eligibility criteria

Inclusion and exclusion criteria can be found in Table 1. Criteria utilized are similar to studies investigating the use of manual therapy treatment procedures with or without exercise in patients with rotator cuff related pain [18, 19, 25–28].

**Table 1:** Inclusion and exclusion criteria

Inclusion criteria	Exclusion criteria
1. Age 18–65 years.	1. Shoulder pain following a traumatic event.
2. Unilateral shoulder pain of atraumatic origin.	2. History and clinical presentation compatible with complete rotator cuff and/or biceps brachii rupture.
3. Scoring at least 3 out of 10 on a numeric pain rating scale.	3. Adhesive capsulitis.
4. Symptoms lasting more than 6 weeks.	4. History of dislocation.
5. Pain on active shoulder movement.	5. Glenohumeral osteoarthritis.
6. Pain provoked by at least three of the following tests: Hawkins-Kennedy, Neer, Painful arc, Empty/full can and Resisted external rotation.	6. Cancer
7. Patients referred by a shoulder specialist with diagnosis of rotator cuff injury (tendinitis/tendinosis), subacromial impingement syndrome, bursitis, subacromial pain, that fulfill the criteria above.	7. Systemic, local or auto-immune inflammatory conditions.
	8. Previous shoulder or neck surgery or fracture.
	9. Familiar pain provoked by neck movements.
	10. Presence of radicular signs.
	11. Use of corticosteroids over the past six months.
	12. Diagnosis of fibromyalgia.
	13. Clinical depression.
	14. Participants under treament for his/her shoulder condition over the last 3 months.

### Interventions

The treatment phase starts after the participant is deemed eligible and agreed to participate voluntarily and signed a structured consent form. After randomization, participants will be allocated to one of two groups described below. The treatment phase will last 5 weeks.

### Experimental group (EG)

#### Exercise programme

The list of exercises to be conducted in all therapeutic sessions (Additional file 1) was constructed based on previous studies and following recommendations commonly reported in the literature [26, 29–31].Two to three sets of 10 to 15 repetitions will be performed using elastic therapeutic bands and dumbells. Three repetitions of 15 s of the stretching exercises will be performed after the strengthening exercises. Exercise progression load will be individually based and managed in a way that a value of a maximum pain score of 5/10 on a verbal rating scale (0 - no pain and 10 - maximal tolerable pain) should be observed during the execution of the exercises. If no such symptom occurs, a score of 6 on a BORG scale (0 – rest and 10 – extremely strong) will be applied. Therefore, during the treatment sessions, the load utilized (dumbells or elastic bands) will be adjusted according to the perception of symptoms. On the first session, 2 sets of 10 repetitions respecting the aforementioned symptoms will be conducted. In this way, participants will become familiar with the exercise programme and this will also inform on immediate symptom reproduction after the session. On the second session, 3 sets of 10 repetitions will be performed. Then, every week after that, 3 sets of 15 repetitions will be conducted with the adjusted load (same load, more load or less load) based on the perception of symptoms. An interval of 45–60 s will be provided between sets and exercises. Participants will be informed about the importance of increasing the load, while still respecting symptoms. In addition, if symptoms provoked by the exercise programme are still present 24 h later, the exercise load will be diminished until this no longer occurs.

#### Mobilisation with movement (MWM)

The participant and physiotherapist will decide on one active shoulder movement more functionally relevant to the individual. Following this, up to four attempts of MWM will be applied to different joints (cervical spine, thoracic region, scapulothoracic, as well as glenohumeral and acromioclavicular joints) and / or in different positions (standing, sitting or lying), in order to identify one particular MWM that improves significantly the shoulder movement previously selected [17]. The shoulder movement will be conducted to the onset of symptoms, should they occur. Then, one set of six to 10 repetitions will be applied repeating the same movement through pain-free range.This process of pragmatically applying MWM will be respected in every session, but from the second session onwards, two to three sets of 10 repetitions will be applied, with an interval of sixty seconds between sets. In case of failure to identify a MWM that improves the movement significantly, the patient will decide which one seems best and one set of six repetitions will be performed to the onset of discomfort.

### Control group (CG)

#### Exercise programme

Exactly the same as the experimental group and conducted in the same way.

#### Sham mobilisation with movement (MWM)

The participant and physiotherapist will decide on one active shoulder movement that is more functionally relevant to the individual. Following this, a sham MWM [18] will be applied and the movement previously selected will be repeated six to ten times in the first consultation. Briefly, the sham condition simulated the MWM procedure with a different hand positioning. The clinician, standing contralaterally to the treated shoulder, will place one hand over the superior aspect of the pectoralis major muscle and the other over the scapula, both hands will make skin contact only without any significant pressure. The participant will be informed that he or she should move to the onset of symptoms, if they occur.This process will be respected in every session, but from the second session onwards, two to three sets of 10 repetitions will be applied, with an interval of sixty seconds between sets. However, in case the sham MWM fails to improve the movement significantly, one set of six repetitions will be performed only**.**

### Outcome measures

#### Primary outcome measures

##### Shoulder pain disability index (SPADI)

SPADI is a self-reported questionnaire that contains 13 different items. There are two domains: pain (5 items) and functional activity (8 items). Each item ranges from 0 (no pain / no difficulty) to 10 (worst imaginable pain / so difficult that requires help). This questionnaire is a valid and well established instrument that helps to discriminate those responding or not to a certain treatment [32]. A reduction of 8–13 points has been reported as being clinically significant [33]. The Brazilian validated version of SPADI will be used [34] at baseline, end of the treatment period, and the final follow-up.

##### Numeric pain rating scale (NPRS)

A NRPS ranging from 0 (no pain) to 10 (worst imaginable pain) is used to measure pain intensity. Scores will be recorded for resting pain, night pain and pain during movement, all related to the previous 24 h. Decreases in pain levels between 1.1 and 2.2 points or a reduction of 32–34% have been reported in the literature as being clinically significant [26, 35]. The scale will be applied at baseline, end of the treatment period and the final follow-up.

#### Secondary outcome measures

##### Active pain-free range of motion (AROM)

AROM will be assessed for flexion, abduction [36], external rotation [37] and hand behind back [38]. All measurements will be conducted to the onset of pain and evaluated by an inclinometer (Baseline® Bubble Inclinometer, Enterprises Inc). Measurements will be taken at baseline and the end of the treatment period. Limitations in AROM might affect the ability to carry out activities of daily living in patients with RCRP [39] and, therefore, determining changes as a result of a treatment programme might be clinically relevant.

##### Pain pressure threshold (PPT)

Measurements will be collected at three different sites: 5 cm distal to the lateral border of the acromion on both sides over the deltoid muscle, and 10 cm distal to the tibiofemoral joint line, over the tibialis anterior muscle on the unaffected side [40]. The importance of having a psychophysical measurement of general mechanical sensitivity is in helping to explore whether there are differences in pain, function, general pain pressure threshold and treatment outcomes in different groups. A calibrated digital algometer (Wagner instruments, model FPX 25) will be utilized for assessments. Three measurements with an interval of 30 s will be taken. PPT will be assessed at baseline and end of the treatment period. This outcome measure will be analysed in a separate publication.

##### Global rating scale of change (GROC)

GROC is a psychometric instrument that assesses the perception of improvement or deterioration from the patient’s perspective [41]. The scale to be used in this study involves a 15 point Likert scale, ranging from − 7 (much worse) to + 7 (completely recovered). Using this scale, the participant will respond to the following question: “Regarding your shoulder problem, how do you assess your shoulder condition since your entry in the study”. Despite evidence of instability in this scale [42], it is important to allow the participant to make an overall assessment of their condition as a result of the treatment delivered. The assessments will be taken at the end of treatment period and at the final follow-up. Previous research have adopted a value of + 5 as a cut-off point to consider that treatment was sucessful [26].

##### Expectations

In health sciences, this construct assesses the beliefs that a patient has in relation to several aspects of implementation and results of therapeutic modalities [43]. Hence, expectations can be positive, negative or neutral. Factors such as a desire that something happens, previous experiences, reports from significant others, are a few of the aspects taken into account when making a prejudgment regarding a therapeutic encounter [44]. Recently, Chester and colleagues [45] investigated multiple putative factors associated with improved function and reduced pain at the end of physiotherapy treatment for people with shoulder pain. One of the strongest predictors found was the patient’s expectation of recovery. Therefore, assessment of expectation in patients with shoulder pain seems important. In this study, the participant will be asked to answer the following question: “How much do you expect your shoulder problem to change as a result of physiotherapy treatment?” A seven point Likert scale ranging from “completely recovered” to “worse than ever” will be used. Commonly, expectations are assessed prior to the start of a treatment programme. In this study, we will assess it at the beginning of the study and after 3 weeks of treatment. We understand that expectation is a dynamic construct that might vary throughout time and its assessment in two different time points may provide important inferences to be made afterwards.This outcome measure will be analysed and reported in a separate publication.

##### Self-efficacy (SE)

SE relates to one’s beliefs that he or she is capable of dealing and executing a certain course of action needed to manage actual and / or prospective events [46]. In health sciences, SE is related to pain and long term incapacity [47], fear of movement [48], and in patients with shoulder pain, is an important factor associated with better therapeutic outcomes [45, 49]. The domains of pain and physical function of the validated Brazilian version of the chronic pain self-efficacy scale will be used in this research [50]. SE will be assessed at baseline, end of the treatment period and on follow-up.

### Participant timeline

**Fig. 2:**
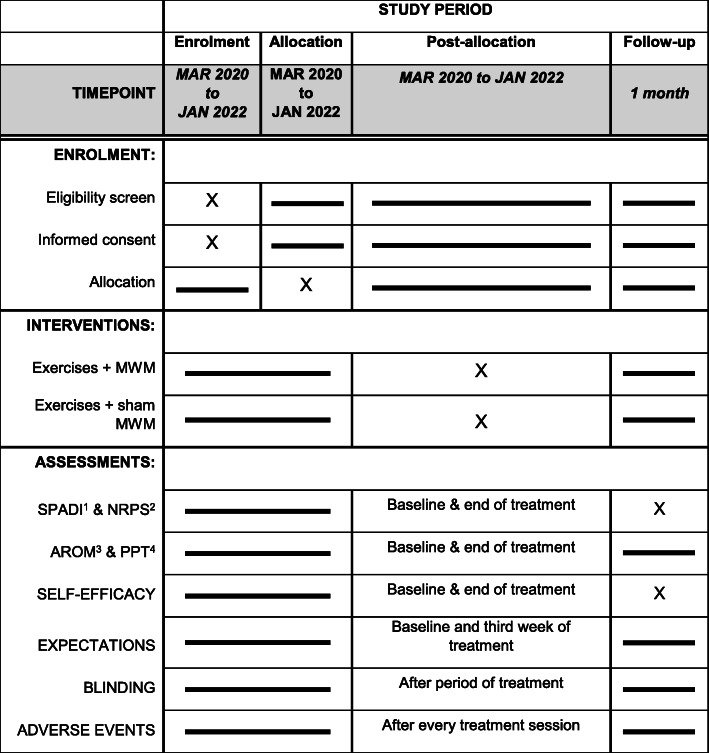
Schedule of enrolment, interventions, and assessments. 1) Shoulder pain and disability index; 2) Numeric pain rating scale; 3) Active range of motion; 4) Pain pressure threshold

The enrollment process will begin from the end of February 2020. After entering the study, participants will have the following outcomes assessed at baseline: SPADI, NPRS, AROM, PPT, expectations and SE; at the end of the treatment period: SPADI, NPRS, AROM, PPT and GROC; and at one month follow-up: SPADI, NPRS, and GROC. Expectation is the only outcome measure to be collected during the third week of treatment. Figure 2 depicts enrolment, interventions and assesssments timeframes throughout the study. Participants will attend ten individual sessions, twice a week (approximately 40 min each), in either of the available treatment sites, according to their geographic locations (see study settings). For the follow-up, participants will be contacted by a research member via email or mail to fill out the respective outcome measures. A telephone call will be made for participants that miss one treatment session without providing explanations.

### Sample size

In order to calculate the number of subjects to be included in this study an alpha value of 0.05 and power of 80% was chosen together with a minimal clinically important difference of 10 points on the SPADI scale with standard deviation of 13.5 points [26]. An initial number of 28 participants in each group was required based on this calculation. With an estimated 20% loss to follow-up, we planned to recruit a total of 70 subjects (35 in each group).

### Recruitment

Subjects will be recruited through a range of strategies. Initially, consecutive patients with shoulder pain seeking treatment at a private physiotherapy practice that agree to participate and fit the inclusion criteria, will be invited to enrol. Second, a research assistant will contact local orthopaedic specialists and inform about the study with an aim to request referrals. Third, study advertisements will be released on social media and a local printed newspaper. Lastly, a partnership established with the health secretary of *São Leopoldo* city will enable subject referral. Therefore, patients fitting the study criteria, referred to the local public physiotherapy service, will be invited to participate.

### Allocation

Participants will be stratified by pain followed by sequence generation, using a computer generated random numbers. In order to reduce predictability of random sequence, blocks of 4 and 6 random numbers will be used. Allocation sequence will be placed in sequentially numbered opaque, sealed envelopes. All allocation procedures will be conducted by a research assistant not involved in any other aspect of the study.

### Blinding

Randomization and group allocation will be performed by a staff assistant not involved in any other aspect of the research. Outcome measures will be collected by a research assistant blind to group allocation, with a formal request not to discuss any aspect of the study with the participants. Data analysis will be conducted by a staff member blind to the nature of the interventions and not engaged in any other aspect of the study. Due to the characteristics of the study, the research assistant conducting the treatments cannot be blinded, nonetheless, this research assistant will not be involved in any of the above procedures. Blinding of participants will be analysed using a three point scale, following specific orientations for this purpose [51].

### Statistical analysis

Data will be analysed using the Statistical Package for Social Science software (SPSS v.20, Inc., Chicago, USA). The analyses will be conducted considering a statistically significant *p*-value ≤0.05. Data normality of the study will be assessed with the Kolmogorov-Smirnov test and homogeneity with the Levene’s test. The results will be reported as the mean with corresponding 95% confidence interval. Intention-to-treat analysis will be conducted so that all patients are analysed within their group allocation. Drop-outs and their reasons will be informed. All data input will be kept in two different files (double data entry) that will be updated every week by a research assistant.

A two-way analysis of variance will be conducted to assess between and within groups differences. For the primary outcome measures (SPADI and NPRS) a 2 × 3 ANOVA with treatment group (EG versus CG) and time (baseline, end of the treatment and follow-up) factors will be performed. Separate 2 × 2 ANOVA will be used for ROM (baseline and end of the treatment period), and a 2 × 3 ANOVA will be conducted for self-efficacy (baseline, end of the treatment period and follow-up). Additionally, appropriate post-hoc tests (Bonferroni) will be used if prior analysis indicates significant differences. GROC analysis will be conducted using descriptive statistics and participants will be classified according to treatment success. Those reporting + 5 or more will be classified as successful. Within groups differences will be calculated at the end of the treatment period and follow-up, and effect sizes will be calculated using Cohen effect size (0.2–0.5: small effect, 0.5–0.8: moderate effect, 0.8 or more: large effect size).

A second study will explore changes in expectation throughout treatment descriptively and whether those differences are associated with SPADI, NPRS and group allocated. Therefore, an ANCOVA will be conducted to analyze the influences of covariates (expectation and self-efficacy) on SPADI and NPRS. A separate 2 × 3 ANOVA will be used to examine PPT measurements in the three different body areas. Post-hoc tests (Bonferroni) will be used if prior analysis indicates significant differences.

### Harms

Due to the nature of this study, we understand that it is important to control for adverse events that might occur as a result of the procedures applied. In order to monitor these, this study will use an adapted questionnaire [52], were participants will respond to the following question: “Have you experienced any discomfort or unpleasent sensation as a result of this treatment?”. Participants will inform (discomfort, soreness, fatigue, etc), rate their sensation using the NPRS (0–10, 10 meaning highest value) and inform when it started (< 30 min after treatment, between 30 min - 4 h, etc) and if it has affected their home or work activities (nothing, little or much). This scale will be applied at each treatment session.

### Research ethics approval, consent & confidentially

Ethical approval for this study was obtained from *Universidade Federal de Ciências da Saúde de Porto Alegre* Ethics Commitee (number 3.528.946) and the trial is registered at ClinicalTrials.gov with the identifier NCT04175184. Table 2 provides information regarding registration data set. Subjects will provide informed consent prior to participation in the trial. Participants personal data and their research data will be kept confidential and will not be disclosed to any other party not participating in the study.

**Table 2:** Trial registration data set

Category	Information
Primary registry and trial identifying number	ClinicalTrials.gov NCT 04175184
Date of registration in primary registry	November, 2019
Ethics Committee number	UFCSPA Ethics Committee CAEE: 3.528.946
Source(s) of monetary or material support	Self-funded
Contact for public queries	Rafael Baeske, rbaeske@yahoo.com
Contact for scientific queries	Rafael Baeske, rbaeske@yahoo.com
Public title	The use of MWM and exercises in shoulder pain.
Scientific title	The inclusion of Mobilisation with Movement to a standard exercise programme for patients with rotator cuff related pain a randomised, placebo-controlled protocol trial.
Countries of recruitment	Brazil
Health condition and problem studied	Shoulder pain related to rotator cuff
Intervention	Mobilisation with movement
Comparator	Sham mobilisation with movement
Key inclusion and exclusion criteria	Age: 18–65 years;Inclusion criteria: ≥6 weeks shoulder pain of atraumatic origin; pain on movement. Exclusion criteria: specific shoulder conditions (fracture, dislocation, arthritis, adhesive capsulitis, cancer, previous surgery, radicular signs).
Study type	Interventional Allocation: randomised; sham-controlled clinical trial with parallel groups; double-blind.
Date of first enrolment	March, 2020
Target sample size	70
Recruitment status	Recruiting
Primary outcome(s)	Function and pain
Key secondary outcome(s)	Active range of motion, pain pressure threshold, global perceived effect, self-efficacy and expectations.

## Discussion

The current evidence for the conservative management of RCRP suggests that exercise with or without manual therapy should be considered. However, despite being recognized as a manual therapy approach, MWM differs from many other manual therapy procedures as it involves active movement on the part of the patient combined with a passive manual therapy procedure. Usually, the active movement chosen is the specific impairment identified as the patient’s main problem. This aspect is particularly important in patients with RCRP as painful and / or restricted movement is commonly encountered on physical examination and subjectively reported as a chief complaint.

Previous studies have found contradictory findings when comparing MWM to sham MWM [18, 19, 21]. The differences in the results observed might be due to methodological aspects (participants´ clinical profile, dosage, type and expertise of the MWM used, follow-up and outcome measures). However, none of the above studies have applied MWM pragmatically. This is considered a critical aspect of the use of MWM. Often in clinical practice, there is a need to change aspects related to the MWM procedure such as: force and direction of the glide, position of the patient, location where the MWM is applied and load used. Therefore, this clinical trial will assist in verifying whether these pragmatic aspects produce better results.

Another key point not sufficiently explored in previous studies is the incorporation of MWM together with exercise in the management of patients with RCRP, reflecting common clinical practice. Only one pilot study has investigated the use of MWM with exercise [22]. However, the age group (83.9 +/− 8.2 years) and clinical settings (nursing home) differs from the current study.

Taking into consideration the high prevalence of RCRP and limited spectrum of studies investigating MWM with exercises, there is a need to verify the impact of adding MWM to an exercise programme in this population. A study comparing different treatment options, that is sham-controlled, will help inform healthcare professionals in the decision making process related to the inclusion or not of MWM in patients with RCRP.

## Supplementary Information

**Additional file 1 Appendix 1**. exercise programme.

## Data Availability

Not yet applicable as the study has not started. However, future data will be available from the corresponding author on reasonable request.

## Abbreviations

ANCOVA: Analysis of covariance
ANOVA: Analysis of variance
AROM: Active range of motion
CONSORT: Consolidated standards of reporting trials
CG: Control group
EG: Experimental group
GROC: Global rating scale of change
MWM: Mobilisation with movement
NPRS: Numeric pain rating scale
PPT: Pain pressure threshold
RCRP: Rotator cuff related pain
SE: Self-efficacy
SPADI: Shoulder pain disability index
SPIRIT: Standard protocol items for randomised interventional trials
SPSS: Statistical package for social Science
