# Supplementary material for: The inclusion of mobilisation with movement to a standard exercise programme for patients with rotator cuff related pain: a randomised, placebo-controlled protocol trial
Source: BMC Musculoskelet Disord. 2020 Nov 12;21:744. doi: 10.1186/s12891-020-03765-6 (PMC7663889; doi:10.1186/s12891-020-03765-6)
Supplement: Supplementary file 1 — Additional file 1 Appendix 1. exercise programme. [file 12891_2020_3765_MOESM1_ESM.zip › APPENDIX 1.docx]

APPENDIX 1

**Exercise programme**


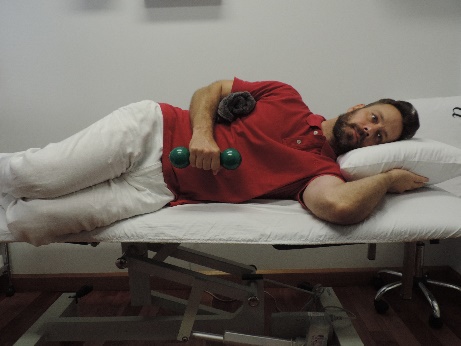

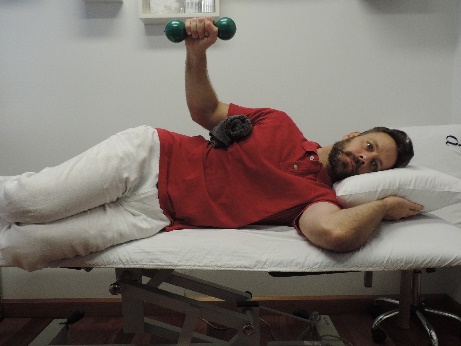


External rotation in side lying


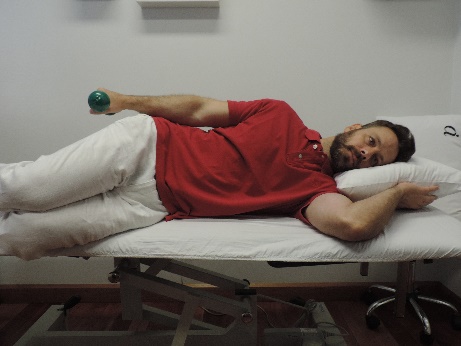

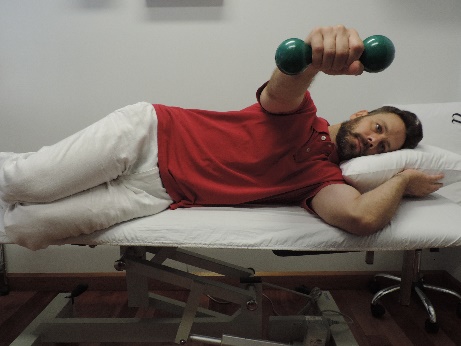


Shoulder flexion in side lying


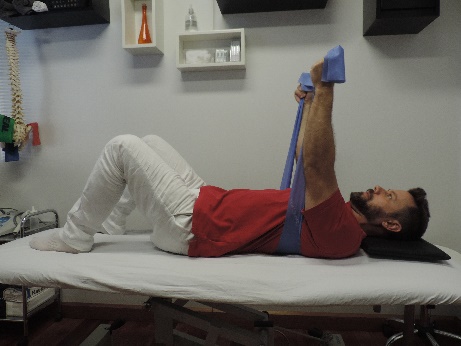

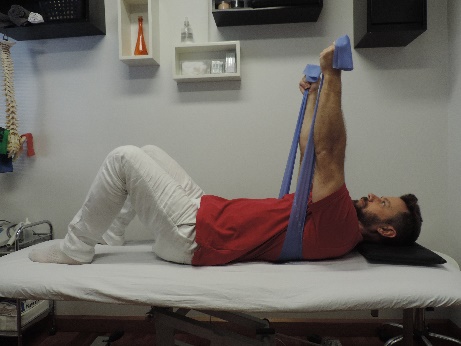


Shoulder protraction in supine


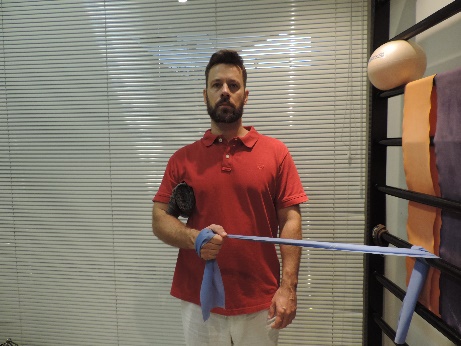

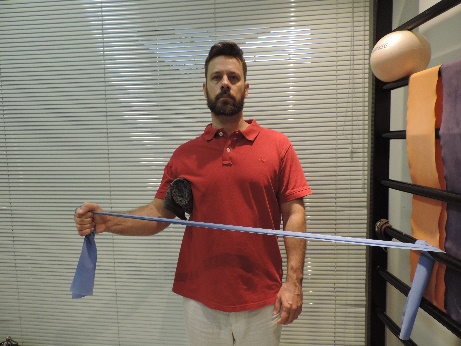


External rotation in standing


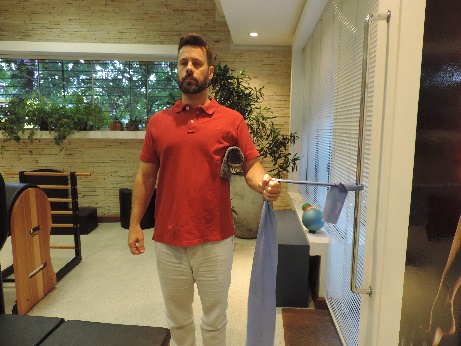

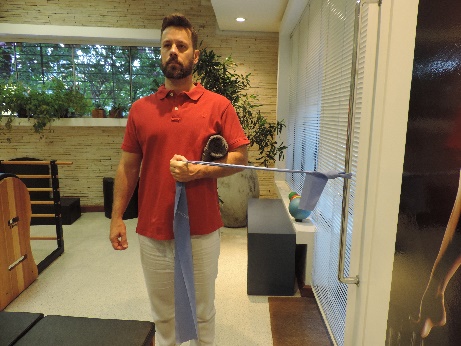


Internal rotation in standing


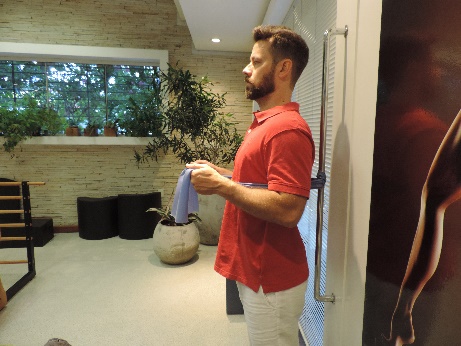

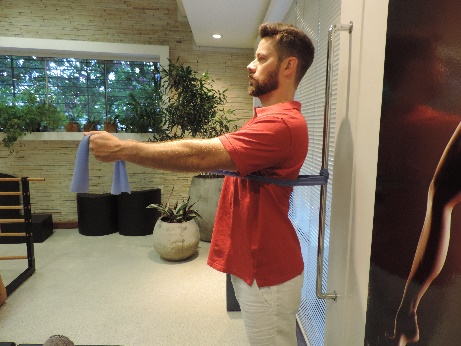


Punch-forwards in standing


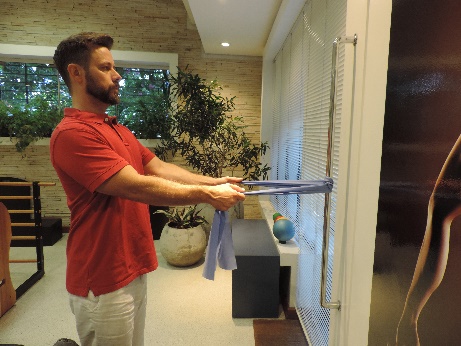

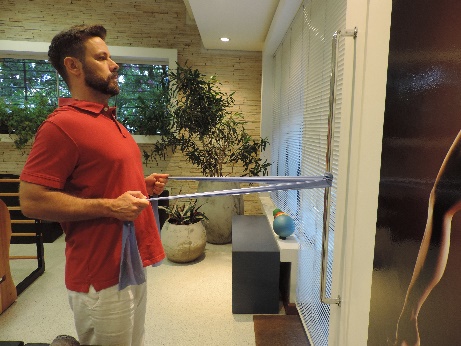


Rowing in standing


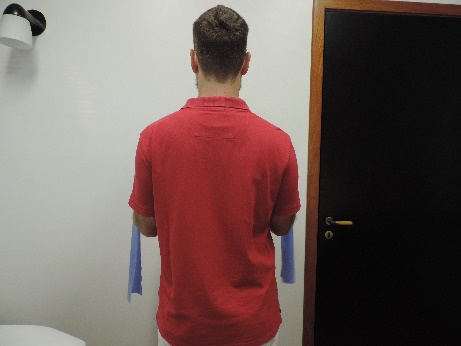

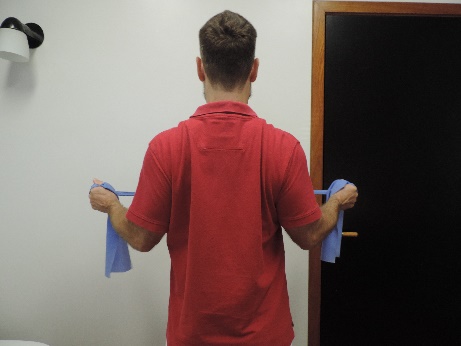


Scapular retraction in standing


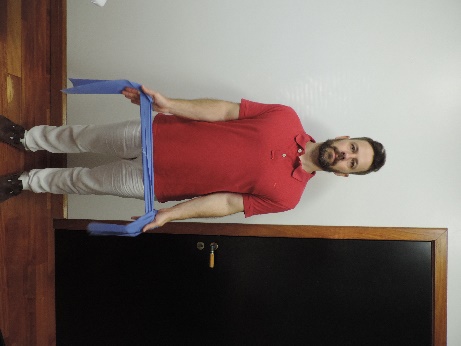

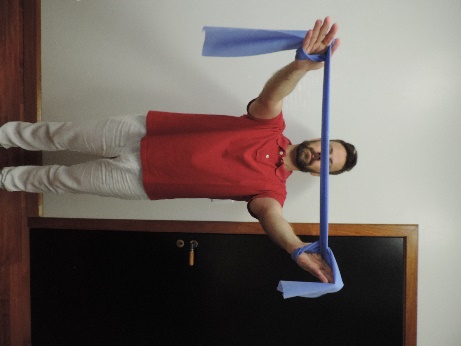

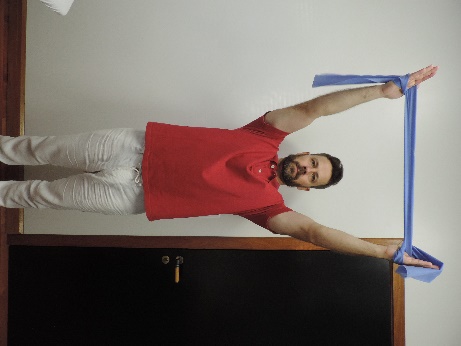


Shoulder elevation with co-contraction of external rotators


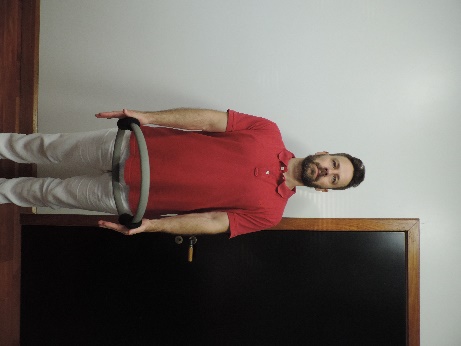

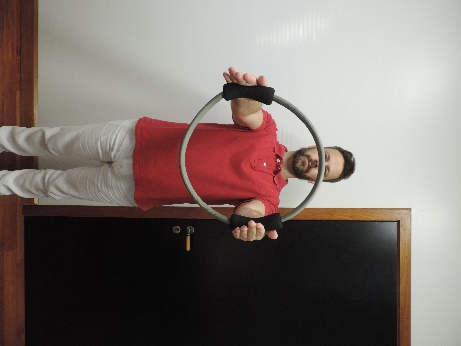

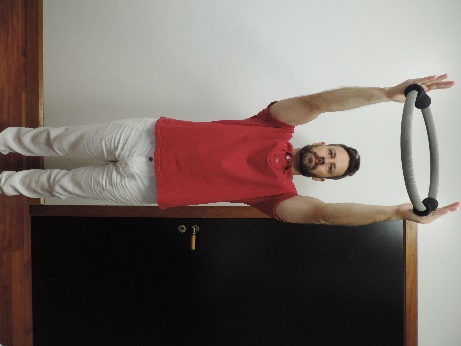


Shoulder elevation with co-contraction of internal rotators


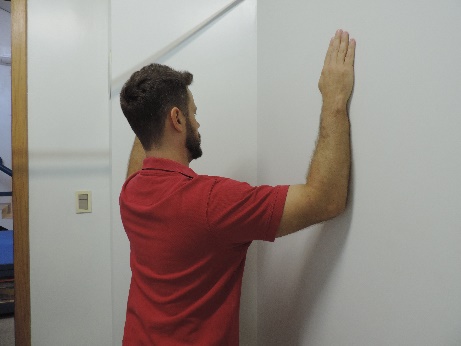

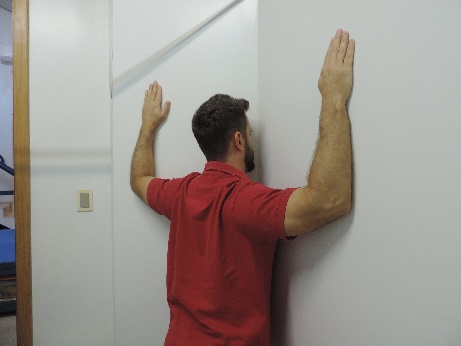


Anterior shoulder stretch


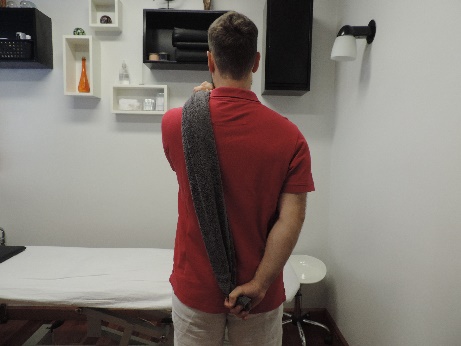

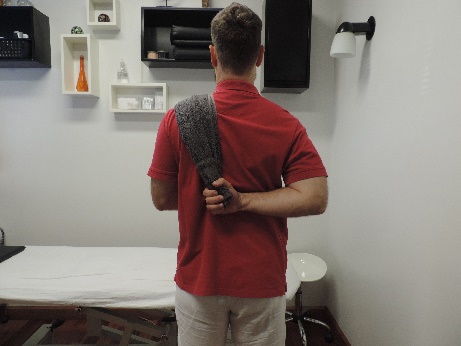


Hand behind back stretch


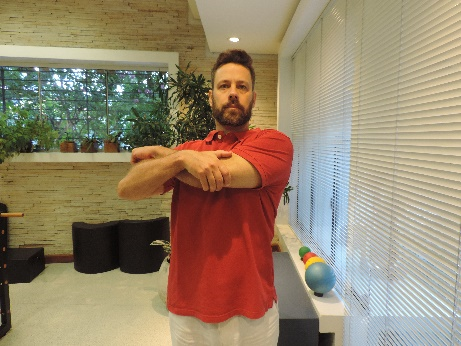


Posterior shoulder stretch
