# Supplementary material for: The inclusion of mobilisation with movement to a standard exercise programme for patients with rotator cuff related pain: a randomised, placebo-controlled protocol trial
Source: BMC Musculoskelet Disord. 2020 Nov 12;21:744. doi: 10.1186/s12891-020-03765-6 (PMC7663889; doi:10.1186/s12891-020-03765-6)

APPENDIX 1

Exercise programme

External rotation in side lying

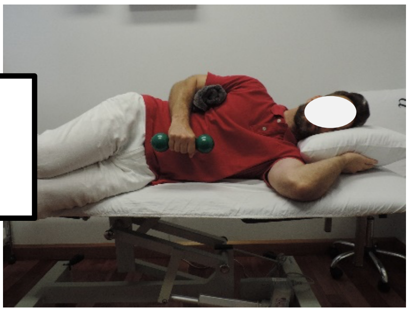

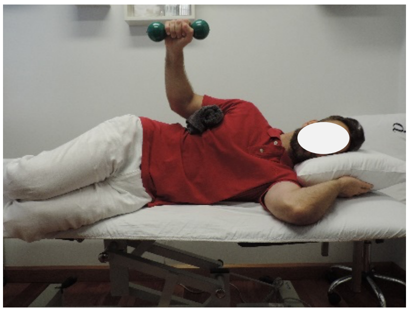

Shoulder flexion in side lying

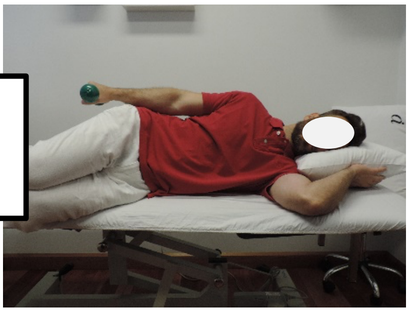

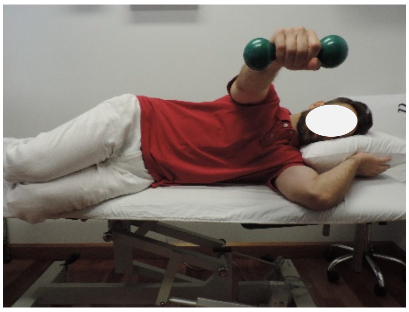

Shoulder protraction in supine

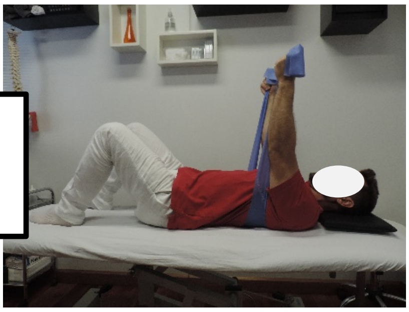

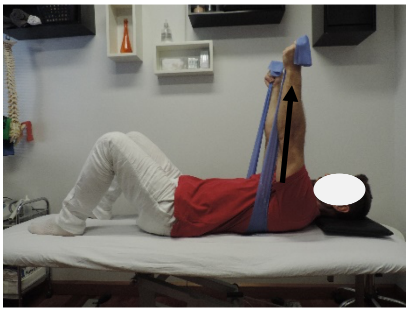

External rotation in standing

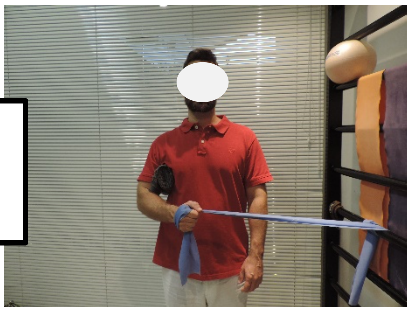

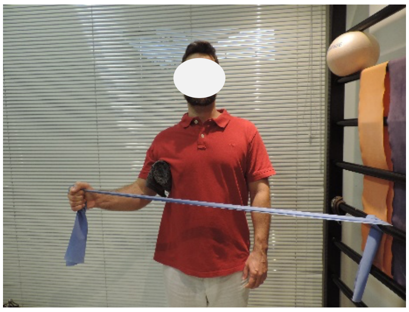

Internal rotation in standing

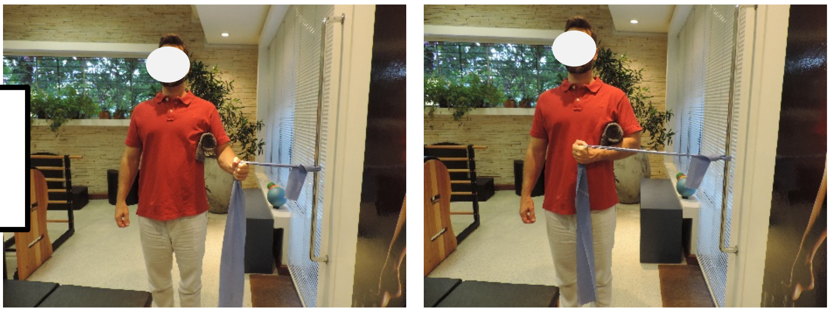

Punch-forwards in standing

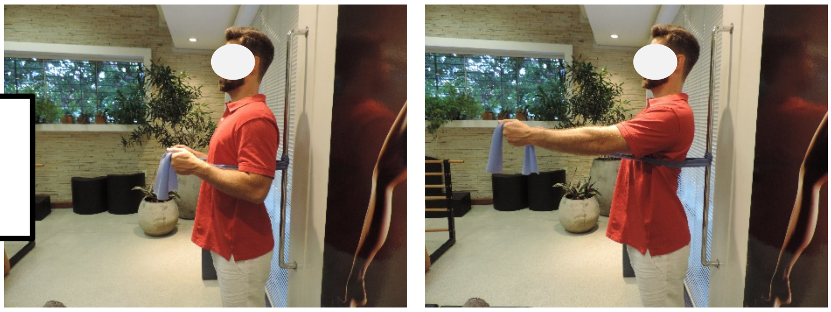

Rowing in standing

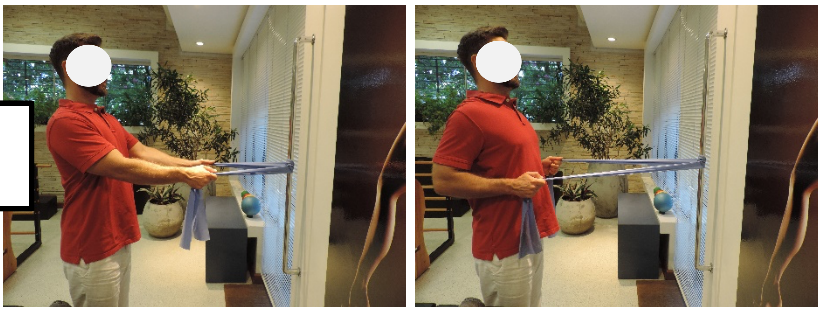

Scapular retraction in standing

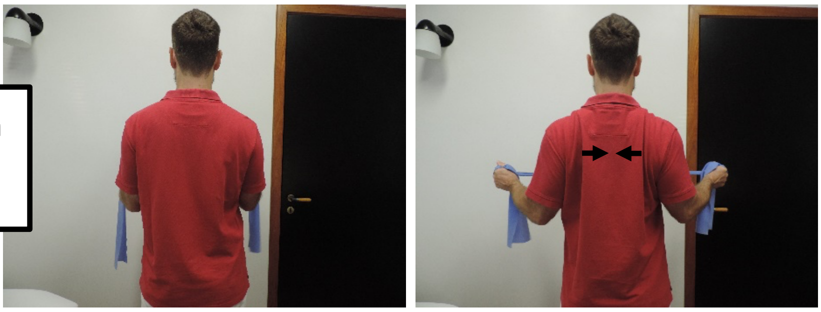

Shoulder elevation with co-contraction of external rotators

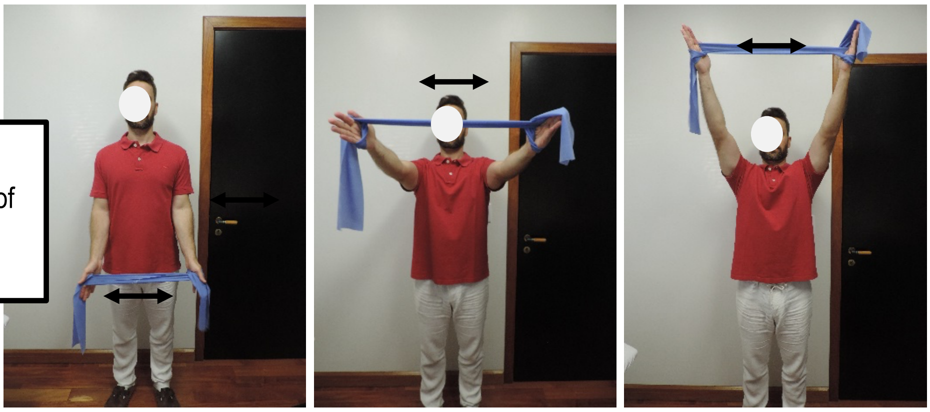

Shoulder elevation with co-contraction of internal rotators

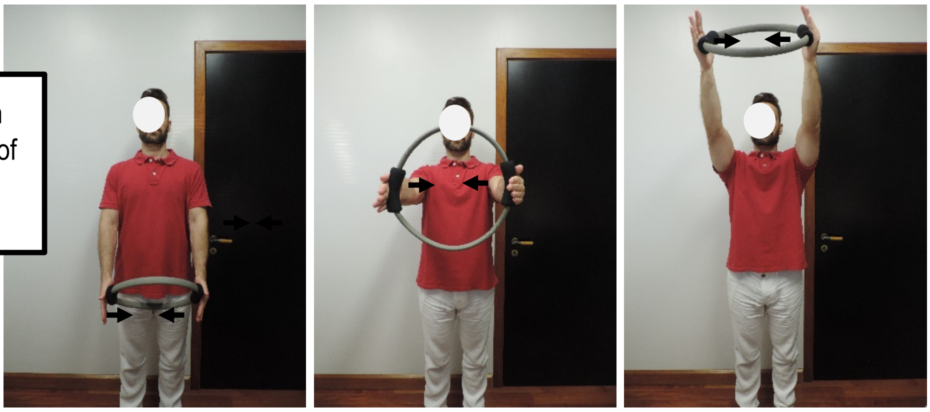

Anterior shoulder stretch

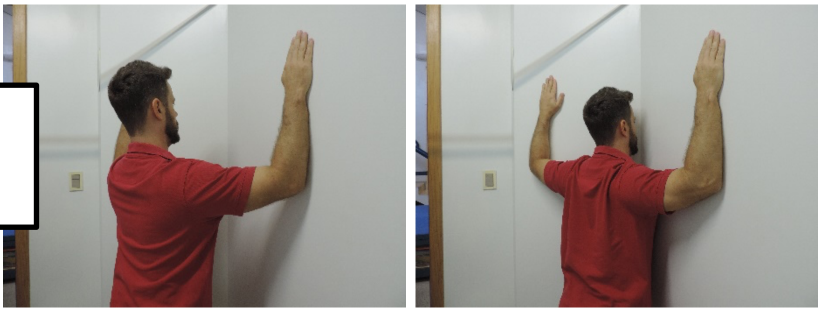

Hand behind back stretch

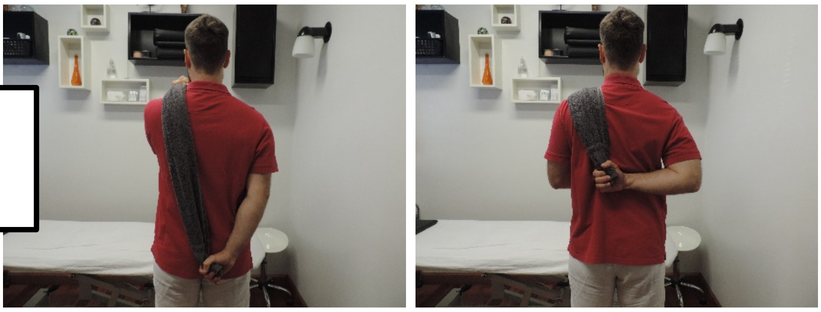

Posterior shoulder stretch

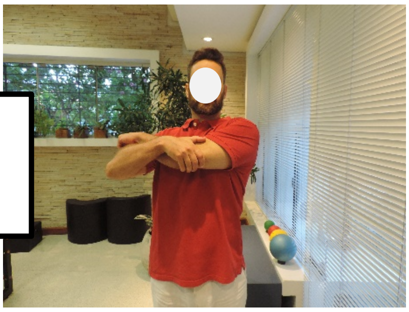

Supplement: Supplementary file 1 — Additional file 1 Appendix 1. exercise programme. [file 12891_2020_3765_MOESM1_ESM.zip › APPENDIX 1.pdf]
